# A three‐dimensional spheroid co‐culture system of neurons and astrocytes derived from Alzheimer's disease patients for drug efficacy testing

**DOI:** 10.1111/cpr.13399

**Published:** 2023-01-11

**Authors:** HyunJung Park, Jaehyeon Kim, Chongsuk Ryou

**Affiliations:** ^1^ Department of Pharmacy College of Pharmacy, and Institute of Pharmaceutical Science & Technology, Hanyang University Ansan Gyeonggi‐do Republic of Korea

## Abstract

Cell culture systems derived from the progenitor cells of human patients have many advantages over animal models for therapeutic drug testing and studies of disease pathogenesis. Here we describe a three‐dimensional (3D) spheroid co‐culture system of neurons and astrocytes derived from induced pluripotent stem cells–neural precursor cells (iPSCs–NPCs) of Alzheimer's disease (AD) patients or healthy individuals that can provide information on drug efficacy unobtainable by 2D co‐culture or monoculture approaches. iPSCs–NPCs of healthy controls or AD patients were seeded onto 96‐well U‐bottom plates and incubated with neuronal differentiation medium for one week and with astrocytic medium for two weeks to replicate the temporal order of cell maturation during brain development. These 3D spheroid models expressed marker proteins for mature neurons and astrocytes. In particular, patient‐derived spheroids showed beta‐amyloid (Aβ) accumulation as revealed by thioflavin T (ThT) staining and ELISA. Aggregation of Aβ induced caspase activation and cell death, while the neuroprotectants nordihydroguaiaretic acid (NDGA) and curcumin (CU) reduced the levels of both ThT and caspase staining. Taken together, these results demonstrate the feasibility of our 3D spheroids combined with ThT and caspase staining as a patient‐based anti‐AD drug screening platform.

## Graphical abstract

(1) Three‐dimensional (3D) spheroids derived from iPSCs–NPCs are promising patient‐based models for efficient drug screening. (2) The 3D spheroids demonstrate remarkably consistent sizes and cellular compositions, indicating their suitability for reliable drug screening. (3) Spheroids derived from AD patient iPSCs–NPCs exhibit stunted neuronal dendrites, Aβ deposits, and reactive astrocytes similar to those in the AD brain, reflecting the disease conditions of patients. (4) ThT and caspase 3 fluorescence staining of spheroids allowed facile detection of changes in Aβ deposition and neuronal cell apoptosis.

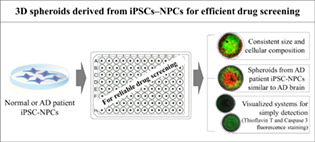

## 1 INTRODUCTION

Alzheimer's disease (AD) is the most common cause of age‐related dementia, and its global incidence is increasing due to the increase in the aging population. AD is characterized by progressive memory deficits and declines in other cognitive abilities that eventually result in loss of functional independence, placing a substantial burden on families and healthcare systems. Therefore, there is an urgent need for drugs that slow or halt AD progression. Robust disease models that recapitulate core aspects of AD pathogenesis are essential for the discovery of effective drugs. Numerous transgenic mouse models harboring mutations associated with familial AD (FAD) have been established; these models exhibit some of the pathological and behavioral signs of the human disease.^1^ For example, transgenic mice with mutations in the amyloid beta (Aβ) precursor protein (APP) and/or presenilin (PS) exhibit the three major pathological hallmarks of the AD brain, namely progressive deposition of extracellular Aβ (beta‐amyloid), intracellular aggregates of hyperphosphorylated tau (pTau) (termed neurofibrillary tangles or NFTs), and associated neuroinflammation. However, the AD‐like neuropathology observed in these models differs in many respects from the human disease, especially the neuroinflammatory response, and changes in gene expression profiles. Therefore, AD drug responses may also differ between mice and humans.^2^

As an alternative to animal models, several research groups have developed three‐dimensional (3D) organoids derived from patient progenitor cells such as induced pluripotent stem cells (iPSCs) expressing FAD‐ or sporadic AD (SAD)‐associated genes.^3^, ^4^, ^5^, ^6^, ^7^ These organoids, which include cultured spheroids, 3D bioprinted brain tissues, brains‐on‐a‐chip, and various combined platforms, reproduce core features of AD pathogenesis, including Aβ plaque deposition, NFT development, and neurodegenerative inflammation. 3D organoids derived from human iPSCs are particularly powerful models for pathological studies and high‐throughput drug screening because they retain the human genetic background (including pathogenic mutations), can be expanded in vitro, and are amenable to a multitude of experimental manipulations and measurement technologies.^8^, ^9^, ^10^, ^11^, ^12^ However, generation of cellular models with sufficient size and cell composition consistency for comparison among trials and that retain the basic cellular organization and gene expression profiles of human brain structures is challenging. Furthermore, these models must be amenable to efficient screening methods for drug testing.

Here, we investigated whether our established human iPSC–NPC‐derived 3D spheroids meet the morphological, functional, and structural requirements for an AD drug discovery platform. We confirmed that our spheroids were composed of mature neurons and astrocytes expressing the expected functional markers after 3 weeks of in vitro culture, were of remarkably consistent size, and were amenable to simple visual screening to assess drug efficacy. These results highlight the potential utility of our 3D spheroid model as a drug screening platform.

## 2 MATERIALS AND METHODS

### 2.1 Lead contact and material availability

Further information and requests for resources and reagents should be directed to and will be fulfilled by contacting the corresponding author. This study did not generate new or unique reagents.

### 2.2 iPSC–NPC lines

The iPSC–NPC lines used in the current study were from normal individuals or AD patients with PS1 mutations (cell line IDs: ax0015 or ax0112). Cell lines were purchased from the AXOL cell line service (East Bush, UK). The experimental use of these iPSC–NPC lines was reviewed by the Institutional Review Board (IRB) of Hanyang University (HYU‐2022‐072), and IRB approval was waived based on this review.

### 2.3 NPC culture

Normal‐ and PS1‐mutated AD‐iPSC–NPC lines were maintained in NPC media consisting of DMEM/F12 supplemented with 1:100 antimycotic‐antibiotics (Welgene, Gyeongsan, Republic of Korea), 1:100 nonessential amino acids, sodium pyruvate (GIBCO, Waltham, MA), d‐glucose (Sigma‐Aldrich, Burlington, MA), l‐glutamine (Welgene), 1:1000 beta‐methanol (Sigma‐Aldrich), 1:50 B‐27 (without vitamin A; GIBCO), 20 ng/ml of basic fibroblast growth factor in a dish coated with poly‐l‐ornithine (Sigma‐Aldrich), and laminin (Sigma‐Aldrich). Accutase (Stem Cell Technologies, Vancouver, BC, Canada) was used to split the cells.

### 2.4 3D neural differentiation of iPSC–NPCs

For 3D neural differentiation of iPSC–NPCs, normal‐ or PS1‐mutated AD‐iPSCs‐NPCs were seeded at a density of 5 × 10^4^ cells in a U‐bottom plate (Thermo Fisher Scientific, Waltham, MA) and then placed in NPC media for 3 days. The medium was changed to neuronal differentiation medium (NDM) on the following day and then every 2 days for 1 week. NDM comprised a 1:1 mixture of DMEM F12: Neurobasal (GIBCO) supplemented with 1× Glutamax (GIBCO) and 1% B27 supplement without vitamin A (GIBCO). Cell culture medium was changed to glial differentiation medium (GDM) on the following day and then every 2 days for 2 weeks. GDM comprised ScienCell astrocyte medium (Cat. 1801, San Diego, CA) supplemented with 1:100 antimycotic‐antibiotics, 1:50 fetal bovine serum, and astrocyte growth supplement (ScienCell Cat. 1852). Spheroids were fixed or subjected to drug efficacy testing at 21 days post differentiation.

### 2.5 Aggregated Aβ preparation and treatment

Aggregated Aβ was prepared as described previously.^13^ In detail, Aβ (1–42) peptide (AGP‐8338, Anygen, Gwangju, Republic of Korea) was suspended in 1,1,1,3,3,3 hexafluoro‐2‐propanol (HFIP; Sigma‐Aldrich) at 1 mM and stored at −20°C until further processing. To form Aβ aggregates, peptides were resuspended to a concentration of 200 μM in dimethyl sulfoxide (DMSO), bath‐sonicated for 10 min, and vortexed for 30 s. Aggregation was allowed to proceed for 72 h at 37°C followed by a 2‐week incubation at 4°C to facilitate higher‐order aggregation. Oligomeric Aβ peptide stock was stored at −70°C until further use. Before use, oligomeric Aβ was diluted in phosphate‐buffered saline (PBS).

Normal iPSCs‐NPCs were incubated with aggregated Aβ (10 μM) in a humidified incubator at 37°C and 5% CO_2_ for 24 h and then fixed for immunocytochemistry and western blotting.

### 2.6 NDGA or CU treatment

For drug efficacy testing of 3D cultured cells, PS1‐mutated AD‐iPSCs‐NPCs were differentiated to spheroids in a U‐bottom plate (Thermo Fisher Scientific). Spheroids were incubated with NDGA (10 μM, Tokyo Chemical Industry, Tokyo, Japan) or CU (10 μM, Tokyo Chemical Industry) in a humidified incubator at 37°C and 5% CO_2_ for 3 days and then fixed for immunocytochemistry and western blotting.

### 2.7 Test tube ThT fluorescence assay

Inhibition of Aβ (1–42) aggregation was measured using the test tube ThT fluorescence assay.^14^, ^15^ Aβ (1–42) stock (0.2 mM) was prepared in 50% DMSO. Stocks of NDGA and CU were prepared in 100% DMSO. Reactions for Aβ (1–42) aggregation (2 μM) and its inhibition by NDGA or CU (10 μM) were performed in 20 mM sodium phosphate buffer (pH 8.0) supplemented with 0.2 mM EDTA and 0.02% sodium azide in the presence of 20 μM ThT in microplate wells (Corning® 96‐well Black Flat Bottom Polystyrene Not Treated Microplate, Corning). ThT fluorescence intensity was recorded every 30 min under 0.5 min shaking at 335 rpm/29.5 min incubation conditions at 37°C using an Infinite M200 pro (TECAN Mannedorf, Switzerland) microplate reader with 450‐nm excitation and 480‐nm emission filters.

### 2.8 Single‐cell analysis for neural development analysis

For single‐cell analysis, a spheroid was dissociated into single cells using Accutase (Stem Cell Technologies). Single cells were analysed by 10× genomics chromium analysis (Macrogen, Seoul, Republic of Korea). The state of neural development (immature neurons, immature astrocytes, mature neurons, or mature astrocytes) was estimated using Loupe Browser 6.0.0.

### 2.9 Real‐time PCR for neural development analysis

Individual spheroids (*n* = 3) were washed with RNase‐free ice‐cold PBS, and total RNA was extracted using TRIzol. Reverse transcription (RT) was conducted using Superscript reverse transcriptase (Invitrogen, Waltham, MA). Total RNA (1 μg) was used as a template for the RT reaction. Quantitative RT‐PCR was performed with the SYBR™ Green system (Thermo Fisher Scientific) using a Light Cycler 480 II system (Roche, Basel, Switzerland). Amplification was monitored and analysed by measuring SYBR green binding. Gene expression was calculated using the C_T_ value, and GAPDH was used as an endogenous control. The forward and reverse primers used in real‐time PCR are shown in Table S2.

### 2.10 Western blot analysis

Five spheroids were lysed with RIPA buffer (150 mM NaCl, 1% Nonidet P‐40, 0.5% deoxycholic acid, 0.1% SDS, and 50 mM Tris–HCl, pH 7.4) containing protease inhibitors (Complete Protease Inhibitor Cocktail, Roche), sonicated for 1 min, and then incubated on ice for 20 min. The supernatant was collected, and protein concentrations were determined using the BCA assay (Thermo Fisher Scientific) after centrifugation at 13,200*g* at 4°C for 15 min. Equal amounts of proteins were then loaded on ~8%–12% SDS polyacrylamide gels. Separated samples were transferred to a PVDF membrane, incubated with primary antibodies against the target proteins, and subsequently incubated with HRP‐conjugated secondary antibodies. Protein bands were visualized by enhanced chemiluminescence (Millipore, Burlington, MA) using WesternBright™ ECL (Advansta, San Jose, CA) and detected using GBox chemi XR5 (Syngene, Cambridge, UK). The relative intensity of each band was measured using ImageJ software (rsb.info.nih.gov). The following primary antibodies were used for western blotting: MAP2 (1:500, Invitrogen), synaptophysin (1: 2000, Abcam, Cambridge, UK), GFAP (1:1000, Dako, Denmark), Kir 4.1 (1:1000, Abcam), component 3 (C3, 1:1000, Abcam), caspase 3 (1:1000, Abcam), and Aβ (6E10, 1:1000, BioLegend, San Diego, CA).

### 2.11 Immunofluorescence

Fixed spheroids were incubated in blocking buffer (10% donkey serum and 0.3% Triton X‐100 in PBS) for 1 day at room temperature. Thereafter, the samples were incubated for 2 days with primary antibodies at 4°C before being incubated with the appropriate fluorescent probe‐conjugated secondary antibodies for 2 days at room temperature while protected from light. Nuclei were stained with DAPI (1:5000; Thermo Fisher Scientific). Images were captured using a confocal microscope (FV3000, Olympus, Tokyo, Japan). The specific primary antibodies used in this experiment were MAP2 (1:200, Invitrogen), GFAP (1:200, Dako), and Aβ (6E10, 1:200, BioLegend).

### 2.12 CellEvent® Caspase‐3/7 Green detection staining

For simple detection of cell death, spheroids were incubated in medium containing 1 μM CellEvent® Caspase‐3/7 Green detection solution (Molecular Probes, Eugene, OR) in a humidified incubator at 37°C and 5% CO_2_ for 24 h or 3 days. After being washed with probe‐free medium, samples were imaged and analysed using fluorescence microscopy (FV3000, Olympus).

Cell penetrance/permeability by CellEvent staining was analysed the confocal z‐stack data to green fluorescence of activated caspase emitted from the inside of spheroids. The confocal z‐stack data were acquired from 100 slides of each 1 μm of one spheroid.

### 2.13 ThT staining of aggregated Aβ in cells

For simple detection of aggregated Aβ, spheroids were incubated with a medium containing 10 μM ThT solution (Invitrogen) in a humidified incubator at 37°C and 5% CO_2_ for 30 min. After washing with probe‐free medium, samples were imaged and analysed using fluorescence microscopy (FV3000, Olympus).

### 2.14 Statistical analysis

Data were analysed using two‐way ANOVAs followed by Tukey's post‐hoc tests or one sample t‐tests using GraphPad Prism (version 5.0; San Diego, CA). Significance was accepted at the 95% probability level. Data are presented as mean ± standard error of the mean.

For calculating the coefficient of variation (CV), we take the standard deviation and divided it by the mean.

$$\mathrm{CV}=\frac{\text{Standard deviation}}{\text{Mean}}$$

The Z factor (*Z*′) value to statistical effect size was calculated using the terms of four parameters: the means (*μ*) and standard deviations (*σ*) of both the positive (p) and negative (n) controls (*μ*_p_, *σ*_p_, and *μ*_n_, *σ*_n_).

$$Z‐\text{factor}=1-\frac{3\left(\sigma_{p}+\sigma_{n}\right)}{\left|\mu_{p}-\mu_{n}\right|}$$

## 3 RESULTS

### 3.1 Establishment of the conditions for a 3D culture system

**FIGURE 1:**
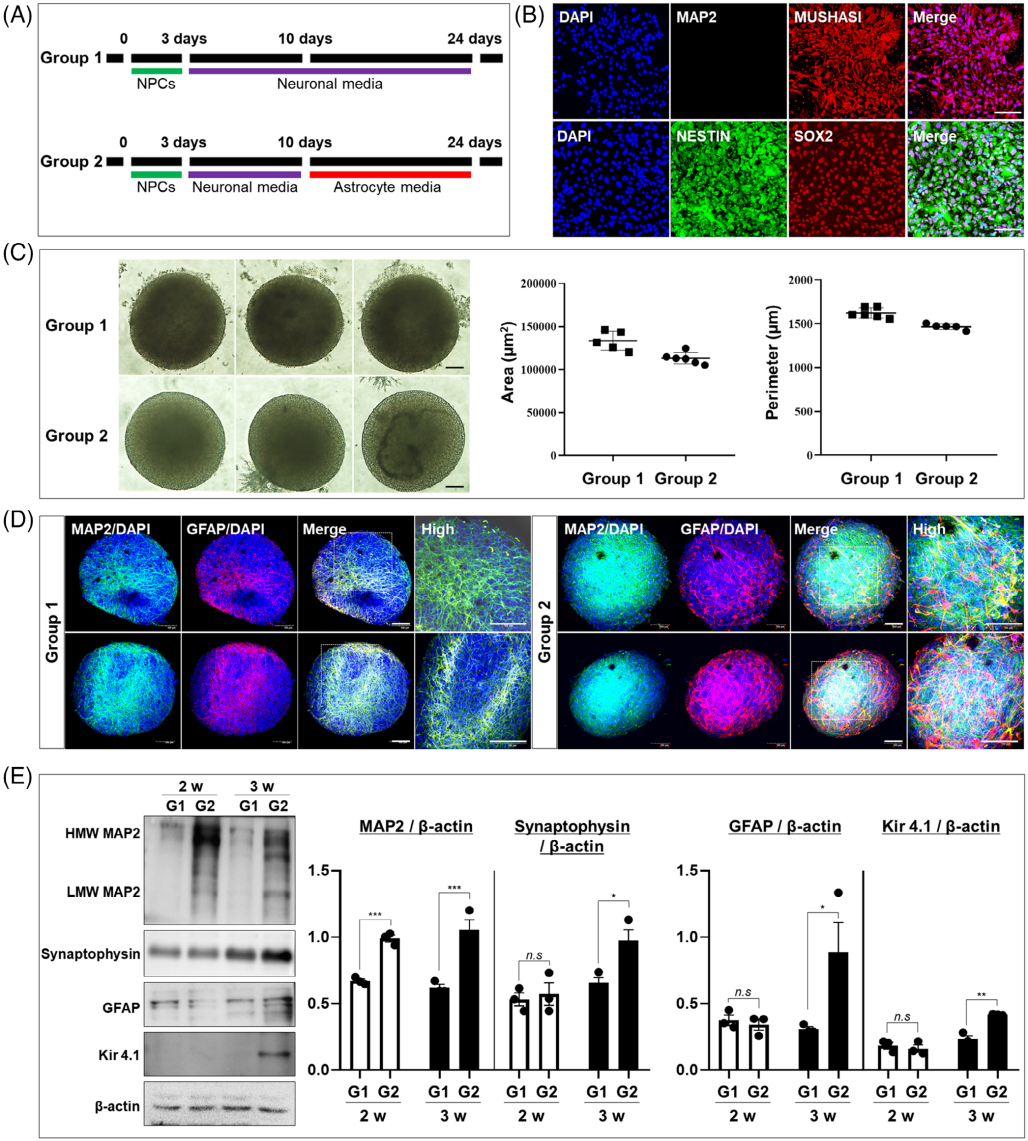
Conditioned medium selection for 3D spheroid co‐culture of neurons and astrocytes. (A) Experimental scheme for iPSC–NPC‐derived spheroids. iPSC–NPCs from healthy individuals were maintained in NPC medium for 3 days. Aggregated cells were cultured under one of two conditions for 3 weeks. Group 1 aggregates were cultured in neuronal medium for 21 days. Group 2 aggregates were incubated in neuronal medium for 7 days and then in astrocyte medium for 14 days. (B) Characterization of spheroid cellular composition by marker expression (positive markers: NESTIN, MUSASHI, SOX2; negative marker: MAP2). (C) Representative images showing Group 1 and 2 spheroids after 24 days in vitro. There were no significant differences in spheroid area and perimeter between Group 1 and 2 spheroids (*n* = 5, data were analysed by one sample t‐test using GraphPad Prism). (D) Neural differentiation of Group 1 and 2 spheroids as assessed by immunostaining for a neuronal marker (MAP2) and astrocyte marker (GFAP). Group 2 spheroids were uniformly surrounded by astrocytes (GFAP), and neurons exhibited robust MAP2‐positive dendrites. (E) Expression levels of the mature neuron markers MAP2 and synaptophysin and the mature astrocyte markers GFAP and Kir4.1 in Group 1 and 2 spheroids as measured by western blotting (*n* = 3 spheroids per group) *p < 0.05, **p < 0.01, and ***p < 0.001 by two‐way ANOVA followed by Tukey's post hoc tests (using GraphPad Prism). Error bars represent the standard error of the mean. All scale bars are 100 μm.

We established a 3D spheroid co‐culture system of neurons and astrocytes using iPSCs–NPCs isolated from healthy controls as illustrated in Figure 1A. Isolated iPSCs–NPCs from healthy donors were cultured under neural differentiation conditions followed by astrocyte generation conditions to replicate the sequence of cell emergence and maturation during brain development.^16^ Healthy control iPSC–NPCs were selected based on positive expression of MUSASHI, NESTIN, and SOX2 and negative expression of MAP2 using immunofluorescence (Figure 1B). The optimal number of seed cells for stable spheroid size was determined to be 50,000 by comparing cultures initiated with 3000, 5000, 10,000, or 50, 000 cells (Figure S1).

To identify a reliable medium for neural differentiation, we first compared spheroids derived from 50,000 cells differentiated using neuronal medium only for 3 weeks (Group 1) or neuronal medium for 1 week followed by astrocyte medium for 2 weeks (Group 2). While we found no difference in spheroid size between these groups (Figure 1C), Group 2 spheroid neurons possessed more numerous dendrites expressing the mature neuronal marker MAP2 and were uniformly surrounded by cells immunopositive for the astrocyte‐specific marker GFAP (Figure 1D). Thus, these spheroids more faithfully recapitulated the cellular organization of the brain than Group 1 spheroids. In both groups, quantification of western blots also revealed expression of the mature neuron markers MAP2 and synaptophysin as well as the mature astrocyte markers GFAP and Kir 4.1 after 2 or 3 weeks of differentiation. After 3 weeks of differentiation, however, Group 2 spheroids showed greater expression levels of MAP2, synaptophysin, and Kir 4.1 than Group 1 spheroids, as well as equivalent expression of GFAP (Figure 1E). Therefore, we used Group 2 culture conditions to establish 3D neuron–astrocyte spheroids from human iPSCs–NPCs.

### 3.2 Characterization of 3D spheroids

**FIGURE 2:**
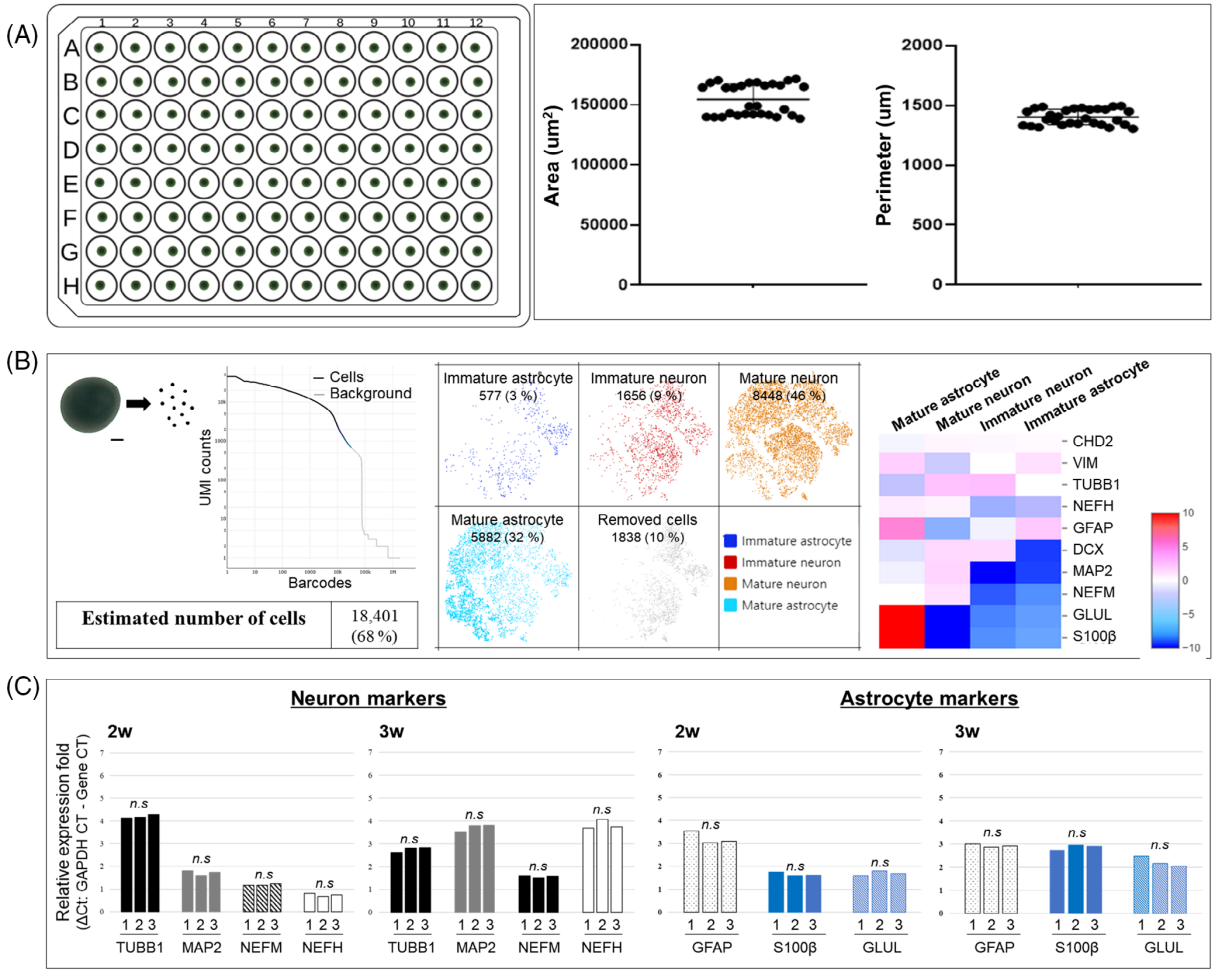
Quantification of spheroid cellular composition by RT‐PCR. (A) iPSCs–‐NPCs (*n* = 50,000/well) from healthy donors (CTL) were seeded in 96‐well U‐bottom plates. After 3 weeks in culture, spheroid area and perimeter were relatively uniform (*n* = 29, one‐sample t‐test using GraphPad Prism). (B) Single‐cell phenotype analysis of an individual spheroid by marker expression profiling. The 18,401 cells consisted of 3% immature astrocytes, 9% immature neurons, 46% mature neurons, and 32% mature astrocytes according to the marker expression heatmap. (C) RT‐PCR analysis of TUBB1, MAP2, NEFM, NEFH, GFAP, S100, and GLUL expression in three spheroids. There were no significant differences in expression levels, suggesting relatively uniform cell phenotype composition (one‐sample *t*‐test using GraphPad Prism).

For drug efficacy testing, spheroids should be relatively uniform in size, cellular composition, and differentiation state. By the end of the sequential 1‐week neuronal and 2‐week glial differentiation protocol, the mean spheroid size was remarkably consistent (area of 154,373 ± 2401 μm^2^ and perimeter of 1403 ± 12.13 μm, *n* = 29) (Figure 2A). The dimension of spheroids was sufficient for immunostaining without clearing and section.^17^

Next, we conducted single‐cell expression profiling by real‐time PCR (RT‐PCR) to assess the consistency of cellular composition and differentiation state. Single cells (~18,401) were isolated from an individual spheroid and expression levels of the immature neuron markers TUBB1 and DCX; immature astrocyte markers CHD2 and VIM; mature neuron markers MAP2, NEFM, and NEFH; and mature astrocyte markers GFAP, S100β, and GLUL were analysed using Loupe Browser 6.0.0 (Table S1). The heatmap of gene expression revealed that a small population of cells expressed relatively high levels of CHD2 and VIM, while another larger population expressed lower levels of CHD2 and VIM but higher levels of S100β, GLUL, and GFAP. In addition, one small population expressed TUBB1 and DCX alone, though the majority of these cells also expressed the mature neuronal markers MAP2, NEFM, and NEFH (Figure 2B). Based on these results, we calculated that each spheroid comprised approximately 3% immature astrocytes, 9% immature neurons, 46% mature neurons, and 32% mature astrocytes. Moreover, RT‐PCR analysis of 3 spheroids revealed no significant differences in TUBB1, MAP2, NEFM, NEFH, GFAP, S100, or GLUL expression among them after 3 weeks of in vitro culture, indicating relatively uniform differentiation among spheroids (Figure 2C). Taken together, these findings suggest that our human iPSC–NPC‐derived 3D spheroids are suitably uniform for drug testing.

### 3.3 Simple detection of cell death and AD pathology

**FIGURE 3:**
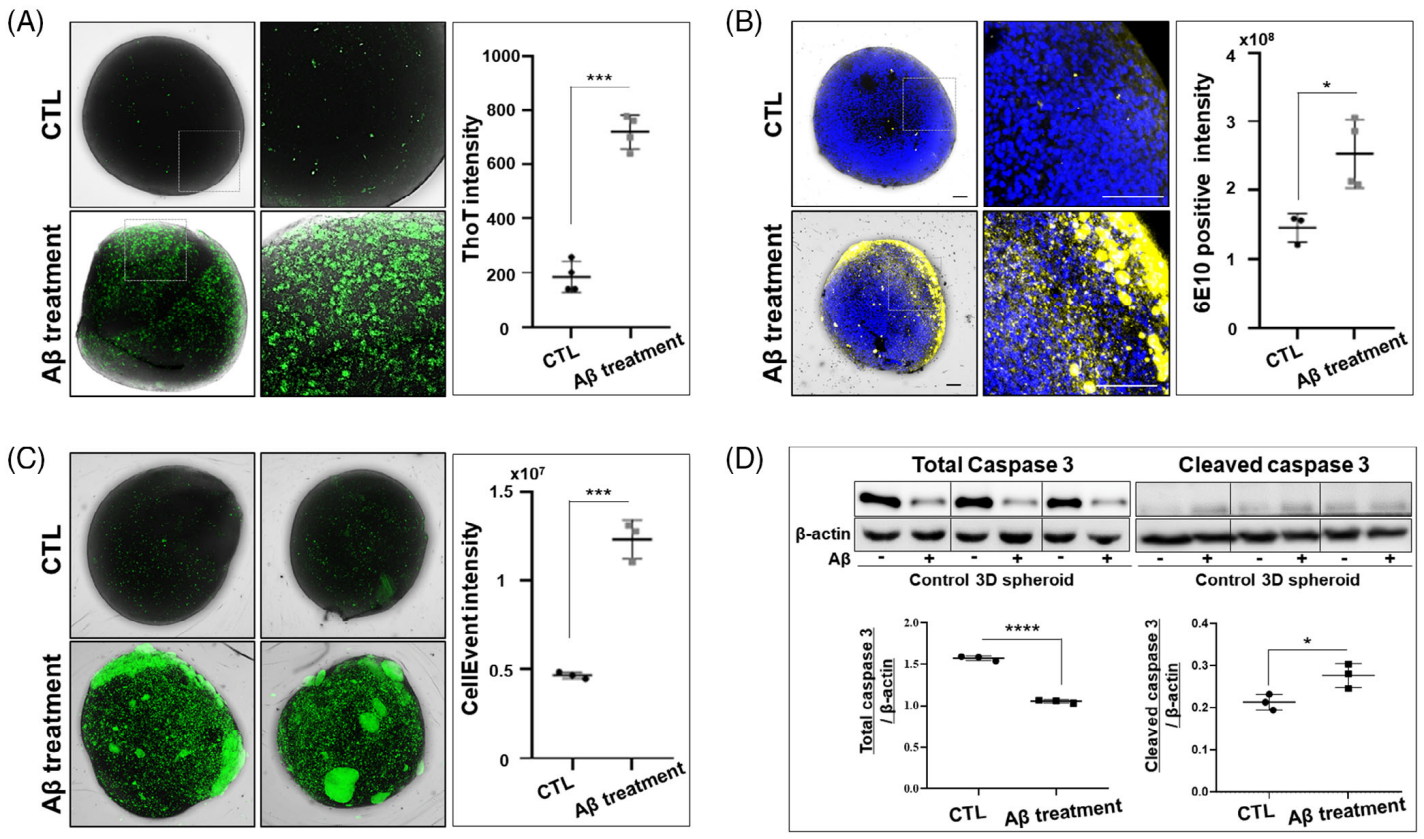
Monitoring Aβ accumulation and apoptosis by ThT and activated caspase staining, respectively. (A) Detection of Aβ aggregates in aggregated Aβ‐treated control 3D spheroids by ThT staining. (B) Detection of Aβ aggregates in aggregated Aβ‐treated control 3D spheroids by Aβ (6E10) antibody staining. Both staining methods revealed similar Aβ distributions. (C) Detection of cell viability and apoptosis in control spheroids using the activated caspase assay. Green fluorescence emission was increased by aggregated Aβ treatment. (D) Quantification of total caspase 3 and cleaved caspase 3 in Aβ‐treated 3D spheroids by western blotting. Expression levels showed the reduction of total caspase 3 level and the increase of cleaved caspase 3 level, compared to untreated 3D spheroids. Data were analysed by two‐tailed t test using GraphPad Prism. Error bars on the bar charts represent standard errors of the mean (*p < 0.05, ***p < 0.001, and ****p < 0.0001). All scale bars are 100 μm.

ThT staining was performed to detect accumulation of Aβ, a major therapeutic target for AD, while CellEvent staining was conducted to detect caspase activation and apoptosis. Exposure of 3D spheroids derived from the iPSCs–NPCs of healthy individuals with oligomeric Aβ resulted in markedly increased ThT fluorescence emission compared to untreated 3D spheroids (Figure 3A). The spatial distribution of ThT fluorescence did not differ substantially from that shown by anti‐Aβ immunofluorescence staining (Figure 3B). Application of oligomeric Aβ also substantially increased the green fluorescence emission of CellEvent staining compared to no treatment (Figure 3C), indicating Aβ‐induced apoptosis. Western blots demonstrated the reduction of total caspase 3 level and the increase of cleaved caspase 3 level, confirming the induction of apoptosis (Figure 3D).

### 3.4 Morphology, neural differentiation state, and pathological state of AD spheroids

**FIGURE 4:**
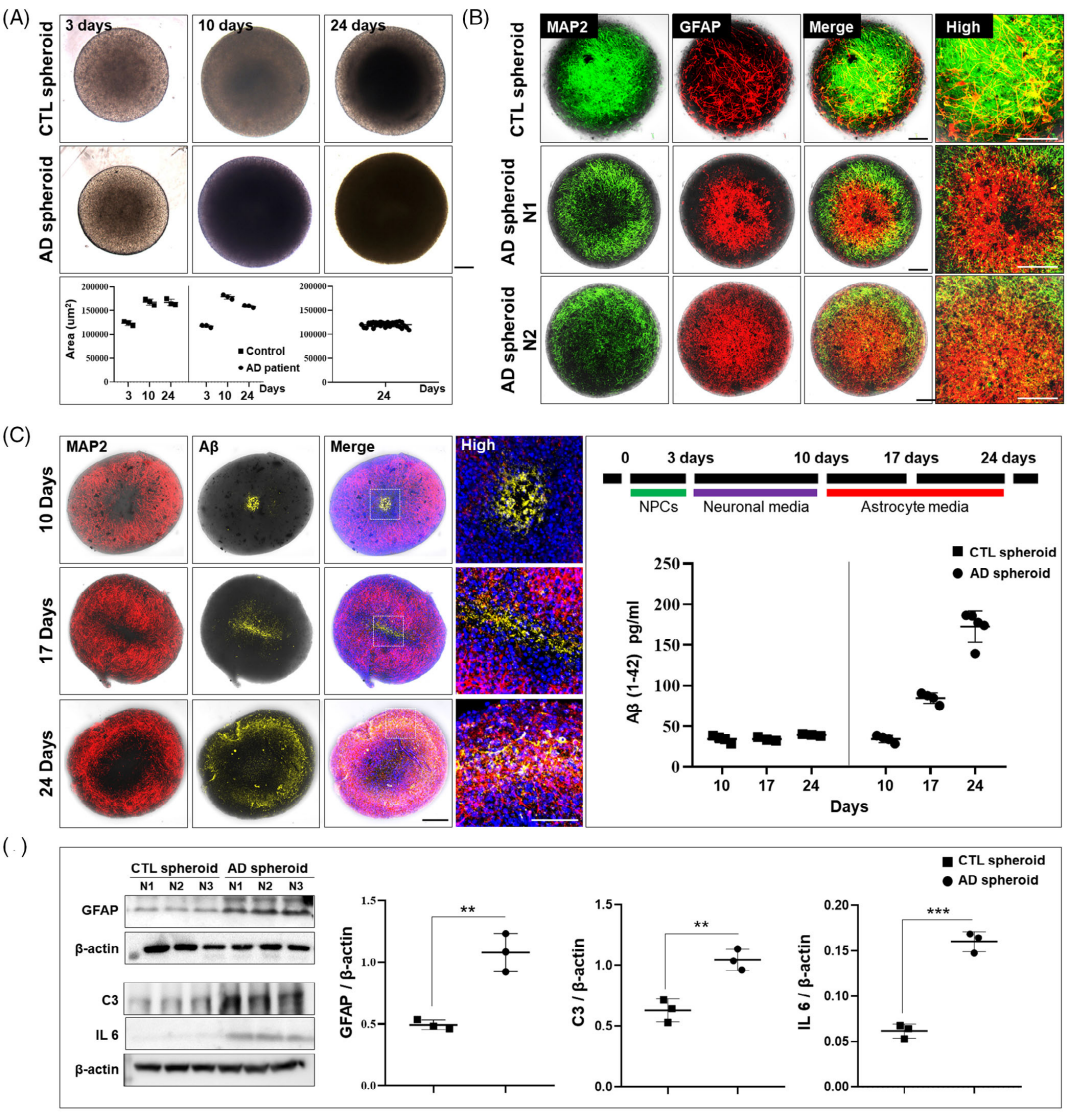
Morphology, differentiation state, and pathology of 3D spheroids from AD patients. (A) 3D spheroids were established from the iPSCs–NPCs of AD patients using the same methods as used for control spheroids (50,000 cells/well in 96‐well U‐bottom plates with a 1‐week incubation in neuronal differentiation medium and a 2‐week incubation in astrocyte differentiation medium). Like controls, AD‐derived spheroids were of relatively uniform size (average of *n* = 36, one‐sample *t*‐test using GraphPad Prism). (B) Differentiation state of AD patient 3D spheroids determined by MAP2 and GFAP immunoexpression. MAP2‐positive dendrites of neurons were shorter than those of control 3D spheroids and GFAP expression was more intense (CTL: Control; N1 and N2: randomly selected independent spheroids). (C) Spheroids of AD patients also showed greater basal immunoexpression of Aβ (stained with Aβ 6E10 antibody) and expression was distributed throughout the spheroid. Quantification of Aβ (1–42) by ELISA. Expression level of Aβ (1–42) was higher than in control 3D spheroids. (D) Quantification of the astrocyte marker GFAP and reactive astrocyte markers C3, IL 6 by western blotting. Expression level of GFAP, C3, and IL 6 was higher in AD spheroids than in control 3D spheroids. Data were analysed by two‐tailed t test using GraphPad Prism. Error bars represent standard errors of the mean (**p < 0.01). All scale bars are 100 μm.

We then repeated these experiments on spheroids derived from the iPSCs–NPCs of AD patients. Like spheroids derived from healthy individuals, those derived from AD patients showed little variation in size (Figure 4A) and pervasive neuronal and astrocytic differentiation as indicated by immunostaining for the same phenotypic markers. However, MAP2‐ and GFAP‐positive cells in AD‐derived 3D spheroids (cell line ID: ax0112) differed morphologically and in their spatial distribution compared to healthy 3D spheroids (cell line ID: ax0015); MAP‐positive cells had shorter dendrites and GFAP‐positive cells had more intense GFAP expression (Figure 4B). AD‐derived spheroids showed progressively increasing Aβ levels based on anti‐Aβ immunofluorescence staining and Aβ (1–42) ELISA. Furthermore, the presence of reactive astrocytes was confirmed by enhanced expression of GFAP, C3, and IL 6 on western blots (Figure 4D). Thus, AD‐derived spheroids exhibited the 3 cardinal pathological characteristics of the AD brain: reactive astrocytes, dendritic degeneration, and Aβ accumulation.

### 3.5 Neuroprotectant‐induced reduction in Aβ deposition and apoptosis in AD spheroids

**FIGURE 5:**
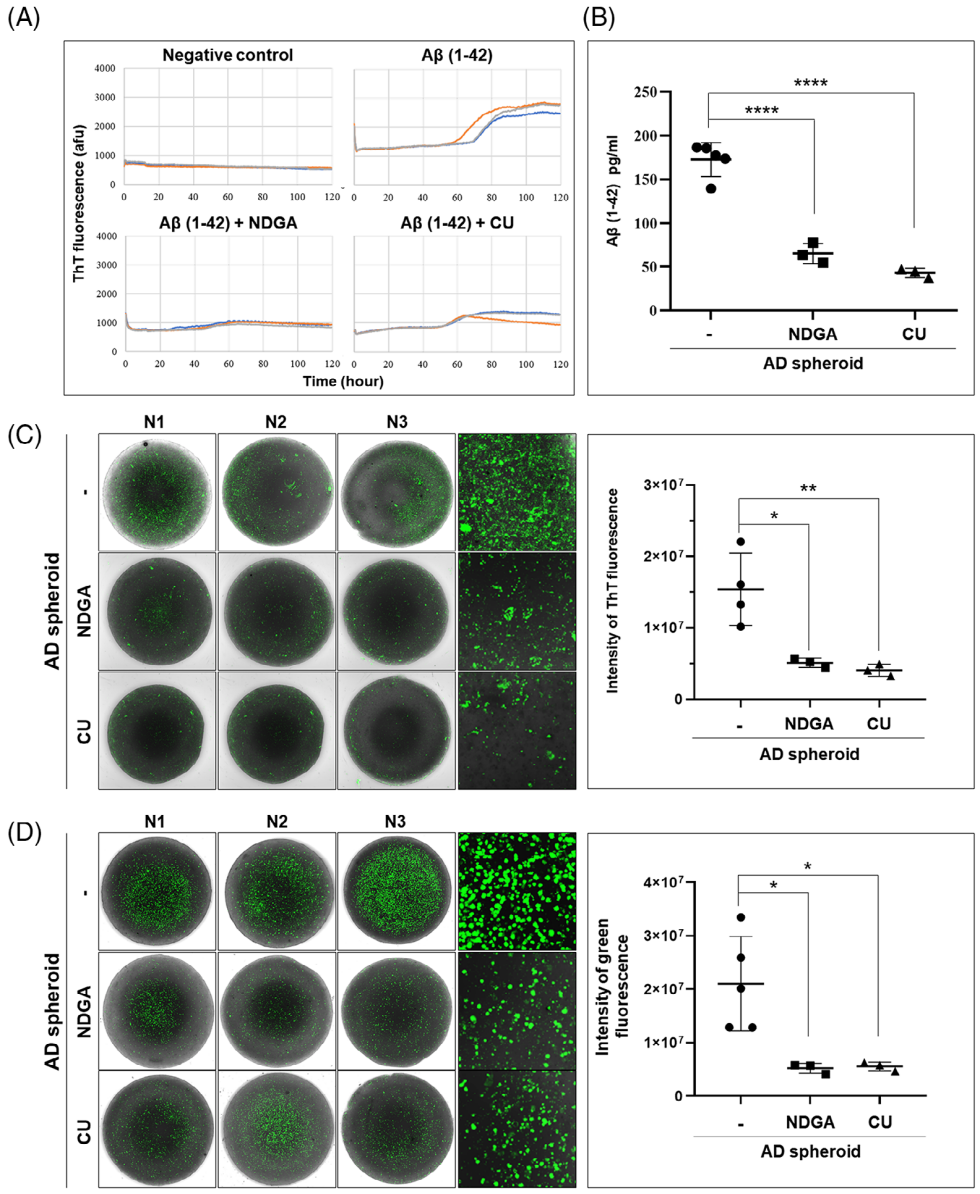
ThT and activated caspase staining of AD patient 3D spheroids for evaluation of anti‐AD drug efficacy. (A) ThT fluorescence assay for Aβ (1–42) aggregation. Supplementation with 10 μM of NDGA or CU prevented the formation of Aβ (1–42) aggregates. (B) Quantification of Aβ (1–42) by ELISA. Incubation of AD‐derived spheroids with NDGA and CU decreased aggregated Aβ (1–42) levels. (C) Changes in Aβ aggregates using ThT staining of AD‐derived 3D spheroids incubated with NDGA or CU. Both NDGA and CU treatments significantly reduced ThT staining intensity in AD‐derived 3D spheroids. (D) Changes in cell death as determined using an activated caspase assay in AD‐derived 3D spheroids incubated with NDGA or CU. Both NDGA and CU treatment also significantly reduced activated caspase apoptosis staining. Data were analysed by two‐way ANOVA followed by Tukey's post hoc tests (using GraphPad Prism). Error bars represent standard errors of the mean (*p < 0.05, **p < 0.01, and ****p < 0.0001). All scale bars are 100 μm. (CTL: Control; N1, N2, and N3: randomly selected independent spheroids).

Finally, we evaluated whether ThT and CellEvent assays in AD‐derived spheroids could identify the drug efficacy of two previously reported anti‐AD compounds, NDGA and CU.^14^, ^15^ In the in vitro ThT fluorescence assay for Aβ (1–42) aggregation, Aβ (1–42) alone demonstrated robust aggregation, while addition of 10 μM NDGA and 10 μM CU to the reaction abolished Aβ (1–42) aggregation (Figure 5A). Based on these data, AD‐derived spheroids were incubated with these compounds. Using ELISA, we demonstrated that NDGA and CU decreased Aβ (1–42) level in AD‐derived spheroids (Figure 5B). In addition, these compounds reversed the increases in ThT and CellEvent fluorescence signals induced by endogenously generated oligomeric Aβ in AD‐derived spheroids, as demonstrated by confocal microscopy. Moreover, the changes in ThT and CellEvent fluorescence signals were qualitatively identical (Figure 5C,D). Thus, both compounds appear to eliminate pathogenic Aβ aggregates and reduce apoptosis. To address the accessibility of compounds from the surface to the centre of spheroids, we examined fluorescence readouts of AD spheroids treated with or without NDGA or CU. The fluorescence intensity and confocal z‐stack images showed the presence of green fluorescence of activated caspase from the surface to the inside of spheroids in AD‐derived spheroids (Figure S2 and Videos [Link], [Link]). Moreover, the reduction of fluorescence as an effect of NDGA or CU also occurred from the surface to the inside of spheroids in AD‐derived spheroids treated with compounds, suggesting cell penetrance/permeability of anti‐AD compounds and staining reagents. Then, to assess the reliability of assays, statistical analyses for the CV and *Z*′ values were performed. The results showed that the CV % was about from 10% to 30%, and *Z*′ value was greater than 0.5 (Tables S3, S4, and S5), suggesting that the assays used in the study is dependable for the screening of anti‐AD agents. Taken together, these findings suggest that our human iPSC–NPC‐derived 3D spheroid model is a pertinent cellular model amenable to simple and easy pharmacological testing to discover novel drugs that show efficacy against AD.

## 4 DISCUSSION

We demonstrated that our iPSC–NPC‐derived 3D spheroid model meets the requirements of consistent size, replicable cell type composition, and amenability to visual screening for high‐throughput drug efficacy testing. Moreover, spheroids derived from the iPSCs–NPCs of AD patients exhibited the core pathologies of AD, indicating their suitability for neuropathophysiological studies.^4^, ^5^, ^6^, ^7^, ^18^ Choi et al. produced 3D cultures of astrocytes and neurons by seeding ReNcell VM human neural stem (ReN) cells harboring FAD mutations on Matrigel for 6 weeks and reported that these 3D culture conditions not only promoted faster and more complete neuronal maturation than 2D cultures, but also that Aβ aggregation and tau isoform levels were increased by 3D culture.^19^ In addition, ReN cells with FAD mutations differentiated into neurons, astrocytes, and microglia on a microfluidic platform. This model also closely recapitulated the core features of AD pathology, including deposition of Aβ plaques, NFT formation, and neurodegenerative inflammation.^6^ However, these models are cumbersome because fabrication of a microfluidic platform is mandatory, and platforms with a constant quality need to be repeatedly generated based on the number of drugs to be tested. Here, we demonstrated that 3D spheroids can be produced reliably with constant size and replicable cell type composition starting from 50,000 iPSCs–NPCs in 96‐well U‐bottom plates. This model fulfils the requirements for testing the efficacy of novel interventions for AD.

We first established culture conditions that recapitulate the sequential differentiation of neurons and astrocytes followed by neuronal maturation and synaptogenesis during brain development. Astrocytes promote synapse formation during development^16^ by synthesizing and secreting various synaptogenic factors.^20^, ^21^, ^22^, ^23^ Neurons cultured in isolation produce few synapses, while synapse formation is markedly increased by addition of astrocytes or astrocyte‐conditioned medium. Several astrocyte‐secreted proteins have been identified to promote the formation of different types of excitatory glutamatergic synapses.^24^, ^25^ As synapse development occurs concomitantly with astrocyte maturation,^16^ iPSCs–NPCs were cultured in astrocyte differentiation medium for 2 weeks following a single week of culture in neuronal differentiation medium to complete astrocyte maturation followed by neuronal maturation and synaptogenesis. Thus, the iPSC–NPC‐derived 3D spheroids described in this study reflect cellular events within the brain.

We then examined if the 3D spheroids that formed after 3 weeks of neuronal and neuronal/astrocytic differentiation expressed markers of mature neurons and astrocytes and whether individual spheroids contained consistent proportions of these cell types. 3D culture conditions have been reported to yield greater proportions of mature neurons and astrocytes than 2D cultures derived from the same ReN cells with FAD mutations.^19^ Similarly, we found that the vast majority of cells in a single spheroid expressed either the mature neuron markers MAP2, synaptophysin, and NEFM or the mature astrocyte markers GFAP, s100β, Kir 4.1, and GLUL. Moreover, the proportions were relatively consistent across spheroids after 3 weeks of culture (*n* = 3).

To directly confirm the suitability of these spheroids for drug efficacy testing, we conducted a simple visual screening assay. Recently, the US Food and Drug Administration approved the monoclonal Aβ antibody aducanumab for AD treatment based on evidence showing that aducanumab reduces Aβ accumulation in the AD brain.^26^ In the current study, we treated our 3D spheroids with two anti‐Aβ aggregation compounds, NDGA and CU, to determine their effects on Aβ aggregation and cell death using visual screening after ThT and activated caspase staining, respectively. ThT fluorescence is widely used for histological staining of protein aggregates.^27^, ^28^ ThT emission has been shown to increase in proportion to Aβ concentration under a number of different experimental conditions,^29^, ^30^ including in live spheroids.^31^ We found that ThT emission was significantly elevated in AD patient‐derived spheroids and control spheroids treated with aggregated Aβ. Furthermore, the spatial distribution of the ThT signal resembled that of immunofluorescence staining using Aβ (6E10) antibody. This emission was also reduced by NDGA or CU treatment, suggesting that these compounds reduce Aβ aggregation. Oligomeric Aβ is known to induce neuronal apoptosis, and there are many fluorescent probes available to monitor apoptosis induction in tissue culture.^32^, ^33^, ^34^, ^35^ For drug screening, we required a dye that would emit fluorescence for prolonged periods as apoptosis may be induced slowly, especially at low Aβ concentration, and reversed slowly by protective agents. Activation of caspase 3 is an essential event in apoptosis induction, and fluorometric detection of caspase activity can be used to assess cell apoptosis in real‐time. Efficient visual screening of apoptosis in cancer cell spheroids was possible over 2 weeks using the CellEvent caspase 3/7 detection kit.^36^ In this study, we detected apoptosis in healthy 3D spheroids incubated with oligomeric Aβ during a 3‐day period and in differentiated, AD patient‐derived 3D spheroids that endogenously produce Aβ. The apoptosis‐associated signal was reduced by NDGA and CU treatment, confirming the usefulness of this approach for visual screening of anti‐AD agents. Taken together, these results suggest that combined use of ThT staining and activated caspase staining of our 3D spheroid models is a reliable strategy to visually assess the effects of drugs on Aβ accumulation and Aβ‐induced apoptosis.

Novel experimental models are essential for elucidating the pathogenic mechanisms of AD and for the development of therapeutics. We generated structurally and functionally homogeneous 3D spheroids from human neural progenitor cells that recapitulate core aspects of AD and that are amenable to visual screening for high‐throughput drug testing based on the alteration of molecular events associated with AD pathophysiology.

## AUTHOR CONTRIBUTIONS

HyunJung Park designed the study, performed experiments, analysed the data, wrote the manuscript, and provided financial support; Jaehyeon Kim performed experiments and analysed the data, Chongsuk Ryou designed and supervised the study, provided financial support, wrote the manuscript, and approved the final manuscript.

## CONFLICT OF INTEREST

None of the authors have competing interests to declare.

## Supporting information


**Data S1:** Supporting Information

**Video S1.** Confocal z‐stack images of activated caspase in AD‐derived 3D spheroid number 1 (N1), Related to Figure 5.

**Video S2.** Confocal z‐stack images of activated caspase in AD‐derived 3D spheroid N2, Related to Figure 5.

**Video S3.** Confocal z‐stack images of activated caspase in AD‐derived 3D spheroid N3, Related to Figure 5.

**Video S4.** Confocal z‐stack images of activated caspase in AD‐derived 3D spheroid with NDGA treatment N1, Related to Figure 5.

**Video S5.** Confocal z‐stack images of activated caspase in AD‐derived 3D spheroid with NDGA treatment N2, Related to Figure 5.

**Video S6.** Confocal z‐stack images of activated caspase in AD‐derived 3D spheroid with NDGA treatment N3, Related to Figure 5.

**Video S7.** Confocal z‐stack images of activated caspase in AD‐derived 3D spheroid with CU treatment N1, Related to Figure 5.

**Video S8.** Confocal z‐stack images of activated caspase in AD‐derived 3D spheroid with CU treatment N2, Related to Figure 5.

**Video S9.** Confocal z‐stack images of activated caspase in AD‐derived 3D spheroid with CU treatment N3, Related to Figure 5.

## Data Availability

The data that support the findings of this study are available from the corresponding author upon reasonable request.
