# Supplementary material for: A three‐dimensional spheroid co‐culture system of neurons and astrocytes derived from Alzheimer's disease patients for drug efficacy testing
Source: Cell Prolif. 2023 Jan 11;56(6):e13399. doi: 10.1111/cpr.13399 (PMC10280145; doi:10.1111/cpr.13399)
Supplement: Supplementary file 1 — Data S1: Supporting Information [file CPR-56-e13399-s004.docx]

**Supplemental Information**

**A 3D spheroid co-culture system of human-derived neurons and astrocytes for Alzheimer’s disease drug testing**

**HyunJung Park ^1,^*****, Jaehyeon Kim ^1^, Chongsuk Ryou ^1,^***

^1^Department of Pharmacological Science, HanYang University, 55 Hanyangdeahak-ro, Sangnok-gu, Ansan, Gyeonggi-do, 15588, Korea

*Co-corresponding authors:

HyunJung PARK, Ph.D.

HanYang University, 55 Hanyangdeahak-ro, Sangnok-gu, Ansan, Gyeonggi-do, 15588, Korea

E-mail: [pphj0105@hanyang.ac.kr](mailto:pphj0105@hanyang.ac.kr)

Chongsuk Ryou, Ph.D.

HanYang University, 55 Hanyangdeahak-ro, Sangnok-gu, Ansan, Gyeonggi-do, 15588, Korea

E-mail: [cryou2@hanyang.ac.kr](mailto:cryou2@hanyang.ac.kr)

**Supplemental Information contains:**

**2 Supplemental Figures**

**Figure S1.**

Selection of the optimal starting cell number for 3D spheroids, Related to Figure 1.

**Figure S2.**

Confocal z-stack intensity of activated caspase in AD-derived 3D spheroids incubated with NDGA or CU, Related to Figure 5.

**5 Supplemental Tables**

**Table S1.**

Gene abbreviation of single cell analysis, Related to Figure 2.

**Table S2.**

Sequences of primer pairs used for target gene amplification in real-time PCR, Related to Figure 2.

**Table S3.**

The coefficient of variation and confidence interval in ThT assay, Related to Figure 5.

**Table S4.**

The coefficient of variation and confidence interval in activated caspase assay, Related to Figure 5.

**Table S5.**

The Z-factor (Z’) values when comparing treatments such as NDGA or CU to AD spheroid, Related to Figure 5.

**9 Supplemental videos**

**Video S1.**

Confocal z-stack images of activated caspase in AD-derived 3D spheroid number 1 (N1), Related to Figure 5.

**Video S2.**

Confocal z-stack images of activated caspase in AD-derived 3D spheroid N2, Related to Figure 5.

**Video S3.**

Confocal z-stack images of activated caspase in AD-derived 3D spheroid N3, Related to Figure 5.

**Video S4.**

Confocal z-stack images of activated caspase in AD-derived 3D spheroid with NDGA treatment N1, Related to Figure 5.

**Video S5.**

Confocal z-stack images of activated caspase in AD-derived 3D spheroid with NDGA treatment N2, Related to Figure 5.

**Video S6.**

Confocal z-stack images of activated caspase in AD-derived 3D spheroid with NDGA treatment N3, Related to Figure 5.

**Video S7.**

Confocal z-stack images of activated caspase in AD-derived 3D spheroid with CU treatment N1, Related to Figure 5.

**Video S8.**

Confocal z-stack images of activated caspase in AD-derived 3D spheroid with CU treatment N2, Related to Figure 5.

**Video S9.**

Confocal z-stack images of activated caspase in AD-derived 3D spheroid with CU treatment N3, Related to Figure 5.


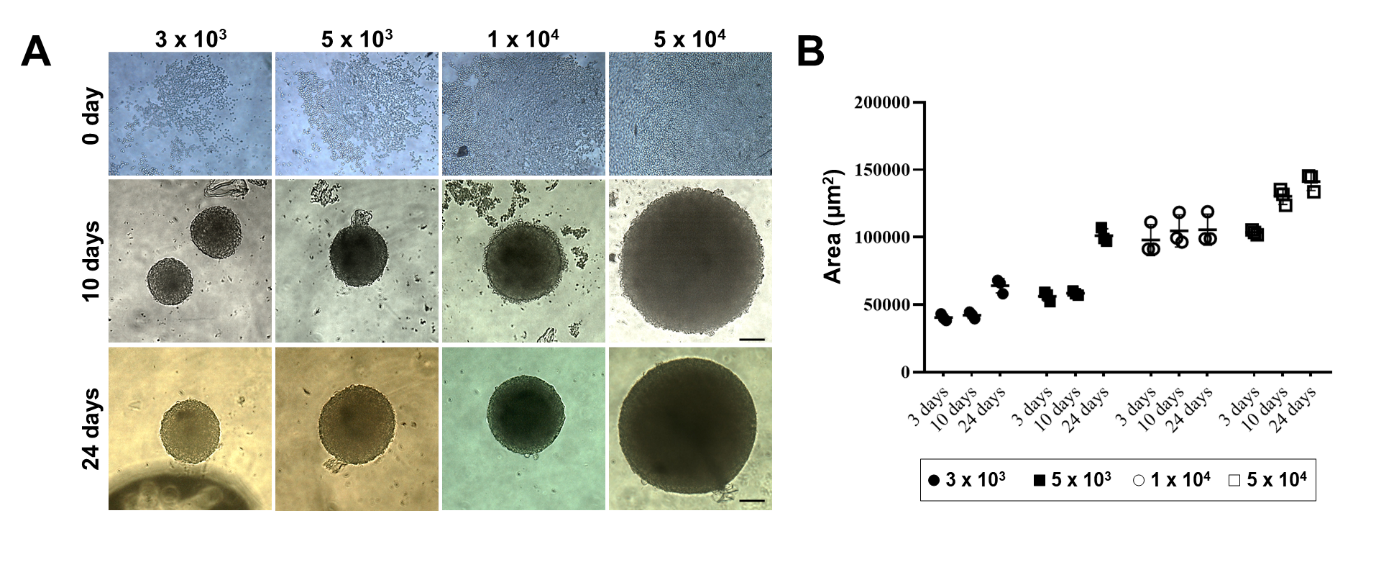


**Figure S1. Selection of the optimal starting cell number for 3D spheroids.**

A. Representative images show the cell aggregates formed by 3000, 5000, 30000, and 50000 iPSCs–NPCs from healthy individuals after 3 days, 10 days, and 24 days in culture (Scale bar: 100 μm). B. Mean spheroid area. Based on these results, we selected 50,000 as the starting cell number for drug testing spheroid generation (n = 3).


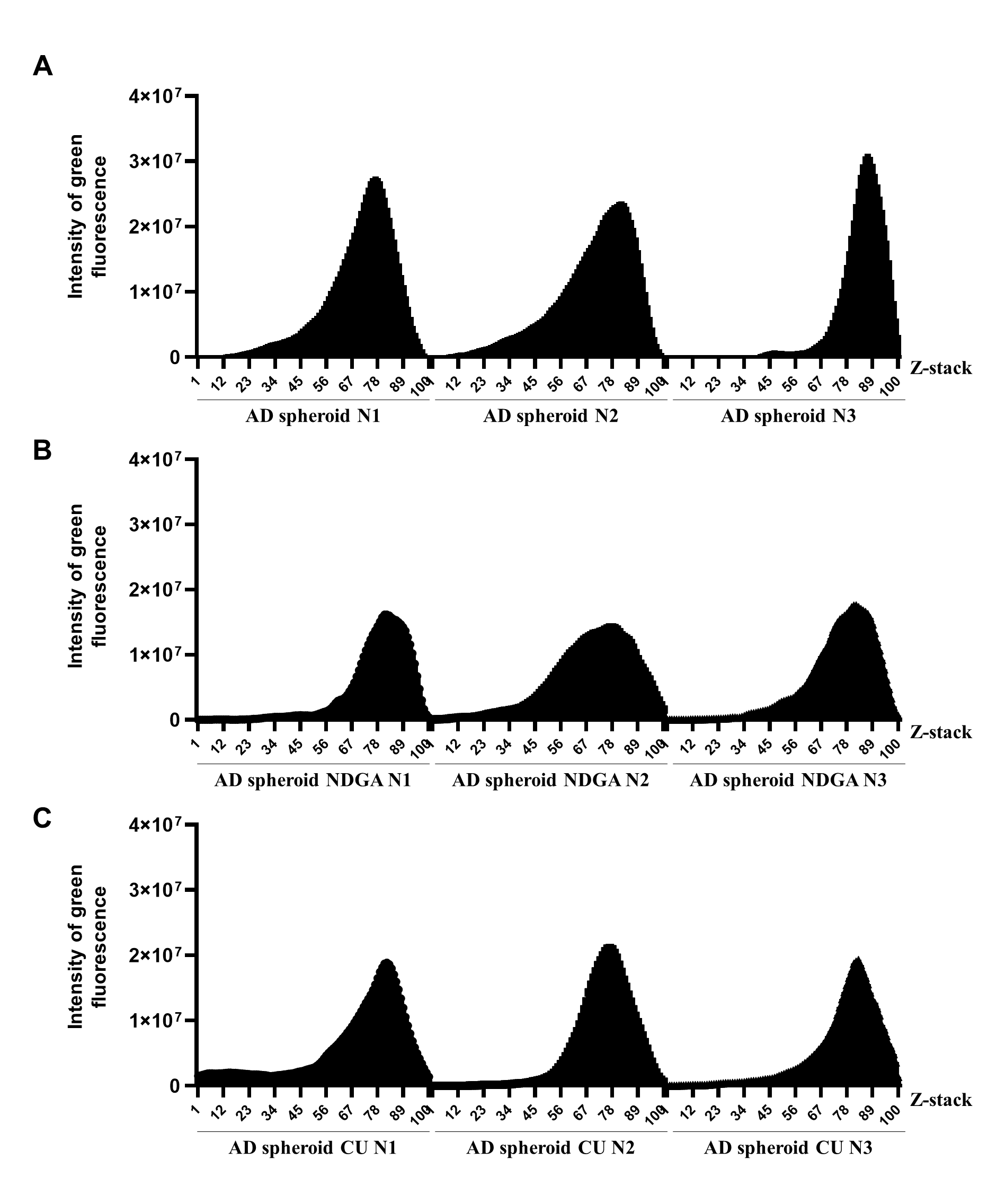


**Figure S2. Confocal z-stack intensity of activated caspase in AD-derived 3D spheroids incubated with NDGA or CU.**

Cell penetrance/permeability by CellEvent staining was identified by intensity of confocal z-stack (μm) images. A.AD spheroid. B. AD spheroid with NDGA treatment. C. AD spheroid with CU treatment.

**Table S1. Gene abbreviation of single cell analysis.**


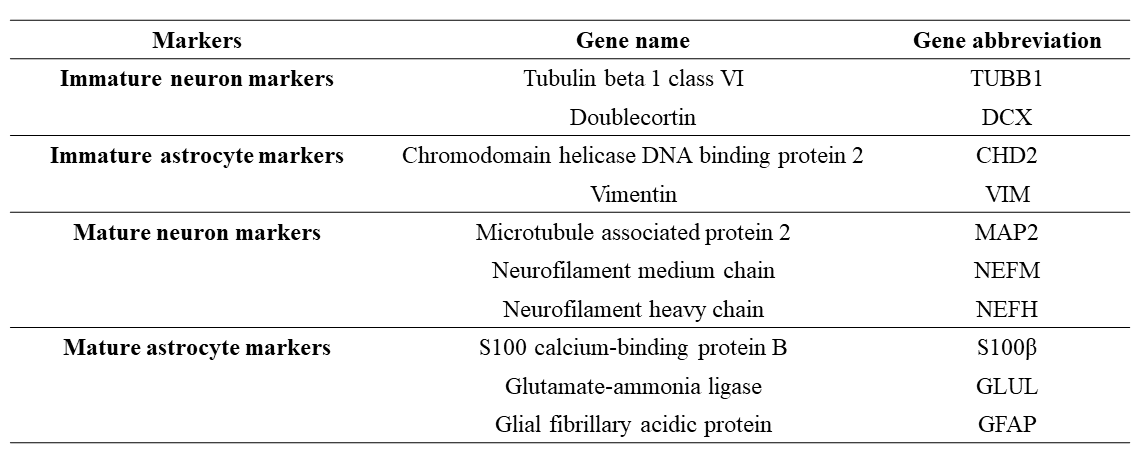


**Table S2. Sequences of primer pairs used for target gene amplification in real-time PCR.**

**
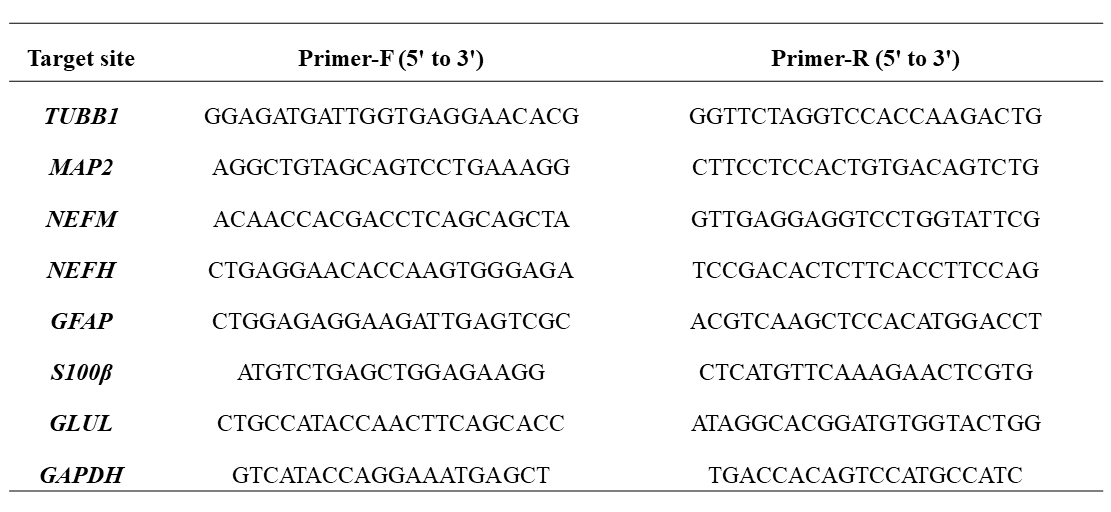
**

**Table S3. The coefficient of variation and confidence interval in ThT assay**

**
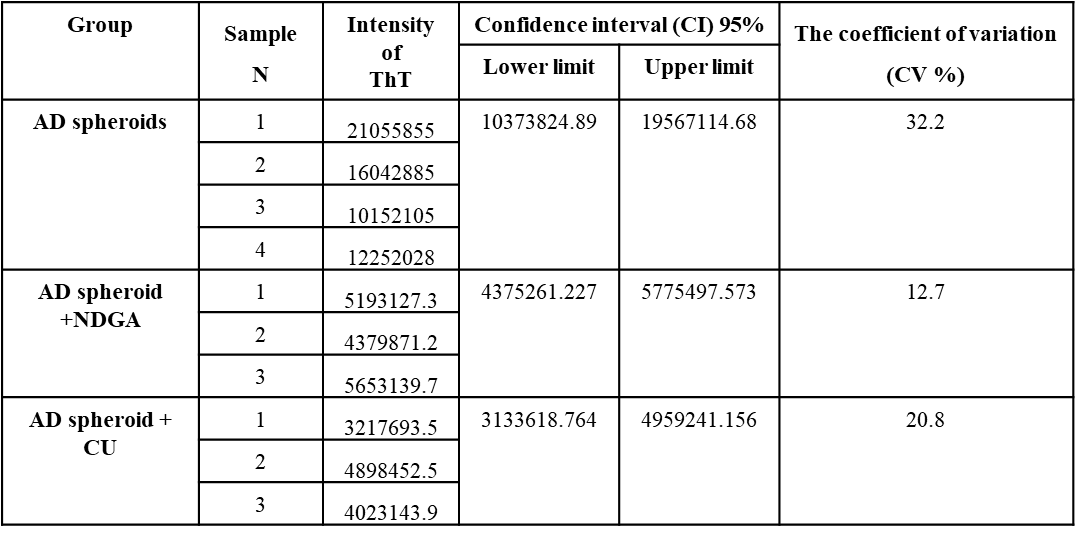
**

**Table S4. The coefficient of variation and confidence interval in activated caspase assay**

**
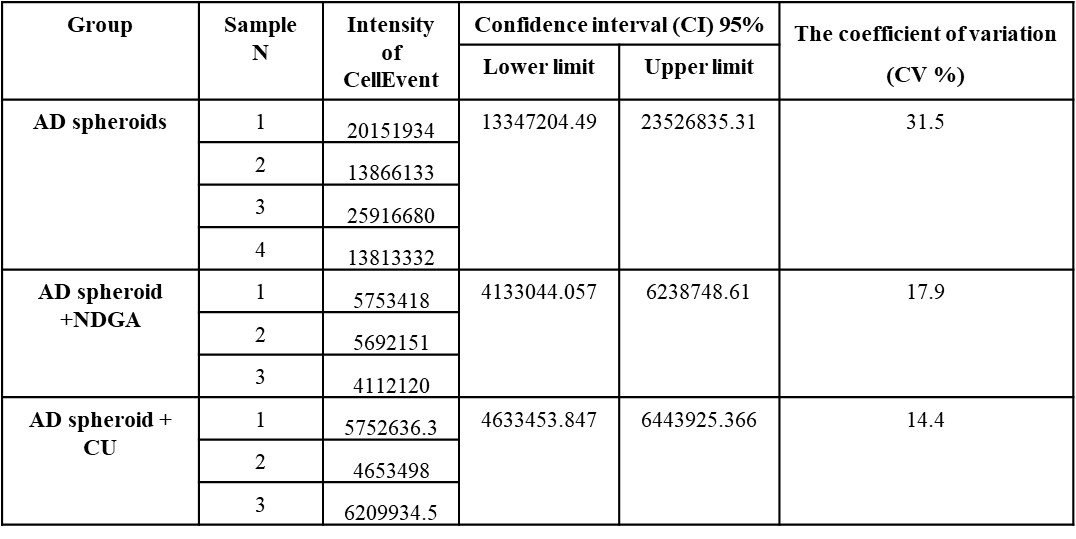
**

**Table S5. The Z-factor (Z’) values when comparing treatments such as NDGA or CU to AD spheroid**

**
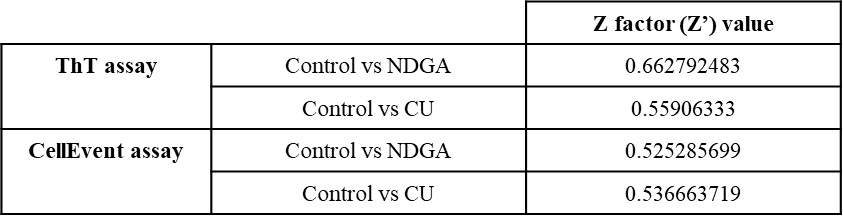
**
